# The Quality of Pre-Hospital Oxygen Therapy in Patients With Multiple Trauma: A Cross-Sectional Study

**DOI:** 10.5812/ircmj.14274

**Published:** 2014-03-05

**Authors:** Mohsen Adib-Hajbaghery, Farzaneh Maghaminejad, Mohammad Paravar

**Affiliations:** 1 Trauma Nursing Research Centre, Kashan University of Medical Sciences, Kashan, IR Iran; 2 Trauma Nursing Research Centre , Medical Surgical Nursing Department, Faculty of Nursing and Midwifery, Kashan University of Medical Sciences, Kashan, IR Iran

**Keywords:** Oxygen Inhalation Therapy, Emergency Medical Services, Multiple Trauma

## Abstract

**Background:**

Trauma is a major healthcare challenge worldwide. In developing countries, most road deaths happen during the pre-hospital phase; consequently, pre-hospital trauma care has received considerable attention during the past decades.

**Objectives:**

The aim of this study was to investigate the quality of pre-hospital oxygen therapy in patients with multiple trauma.

**Patients and Methods:**

This cross-sectional study was conducted in the year 2013. The study population consisted of all patients with multiple trauma who had been transferred by emergency medical services to the central trauma department in Shahid Beheshti Medical Center, Kashan, Iran. The data collection instrument had three parts including demographic, a trauma assessment, and an oxygen therapy quality assessment questionnaires that were designed by the researchers. In total, 350 patients with multiple trauma were recruited from March through July 2013. Data were described by using frequency tables, central tendency measures, and variability indices. Moreover, we analyzed data by using the Chi-square test, Mann-Whitney U test, and the logistic regression analysis.

**Results:**

The study sample consisted of 263 (75.1%) male and 87 (24.9%) female patients. Overall, 211 patients needed oxygen therapy during the pre-hospital phase; however, only 35 (16.60%) patients had received oxygen. The quality of oxygen therapy was undesirable in 92.42% of cases. In addition, 83.4% of patients, whose pre-hospital records indicated the administration of oxygen, reported that they had not received oxygen therapy. Logistic regression analysis revealed that the place of accident and the level of patients' education were significant predictors for administration of oxygen during the pre-hospital phase (P < 0.001).

**Conclusions:**

The quality of pre-hospital oxygen therapy had been provided for the patients with multiple trauma was poor while these patients, particularly patients with chest traumas and head injuries, were in urgent need of oxygen therapy. Consequently, developing and implementing standard evidence-based oxygen therapy protocols and administrating continuous education programs are recommended.

## 1. Background

Trauma is a major healthcare challenge worldwide (1, 2). It causes more than 50 million deaths and 100 million cases of disability each year (3). In Europe, nearly 800000 people die from injuries every year (4). Road traffic injuries are considered the third greatest cause of mortality after myocardial infarction and cerebrovascular diseases. Iran has one of the highest road traffic mortality rates in the world that accounts for over 27000 deaths and almost 0.8 million injured people, which is equal to 1.1 percent of the population (5). Most of these injuries occur between the ages of 20 and 30 years (6). Road traffic accidents are a major cause of traumas and injuries and are estimated to become the third leading cause of death by 2020 (7, 8). In developing countries, most road deaths happen during the pre-hospital phase (9). Bahadori et al. reported that 50% of road deaths happened during the first hour after the accident, 25% during transferring patient to hospital, and 25% during hospital stay and secondary to nosocomial infections (10). Consequently, pre-hospital trauma care has received considerable attention during the past decades (4).

Emergency Medical Services (EMS) is an important part of healthcare services (11, 12). The most important aims of EMS are providing pre-hospital care to trauma patients, stabilizing their vital signs, preventing additional traumas and injuries, preventing death and disability, and transferring them to hospital settings (13, 14). A key pre-hospital measure has been provided to trauma patients is oxygen therapy. During the pre-hospital phase, trauma patients need to be ventilated with 100% oxygen (15). Oxygen therapy improves cellular metabolism (16) and hence, decreases the mortality rate in patients with multiple trauma and head injury (17). Narotam et al. reported that oxygen therapy significantly decreased mortality rate and improved patient outcomes in patients with severe head injuries (18). Prakash et al. investigated the effects of hyperbaric oxygen therapy administered to children with head injury and found that children in the experimental group had significantly shorter hospital stay and fewer long-term complications (19).

In 2007, the Iranian Government introduced the National Pre-hospital Emergency Medical Services Act. Accordingly, the Iranian Ministry of Health and Medical education have recognized the necessity of improving the quality standards for pre-hospital care (3). Evaluating the quality of pre-hospital oxygen therapy is an important component of assuring pre-hospital care quality. However, to the best of our knowledge, the quality of pre-hospital oxygen therapy is poorly evaluated. Barsuk et al. found that 20% of patients with chest trauma and 19% of patients with head injury did not receive pre-hospital oxygen therapy (20). The only study on the quality of pre-hospital EMS in Iran revealed that 20% of trauma patients did not receive oxygen therapy (21). Rood-Dehghan et al. also conducted a study on practicing hospital staff nurses and reported that their performance on oxygen therapy was weak (22).

## 2. Objectives

The aim of this study was to investigate the quality of pre-hospital oxygen therapy in patients with multiple traumas.

## 3. Patients and Methods

This cross-sectional study was conducted from March through July 2013. In order to calculate the sample size, registered data concerning the number of multiple trauma patients in the same period of the previous year was obtained from the archives of the emergency department in Shahid Beheshti Medical Center and the records in the pre-hospital EMS in Kashan, Iran. Based on the recorded data, 325 patients with multiple traumas have been registered at the same period of the previous year. Then, the number of samples was estimated to be about 325 patients. However, 350 patients with multiple traumas were referred during the present study.

The study population was consisted of all patients with multiple trauma who had been transferred by EMS to the trauma center of Shahid Beheshti Medical Center, which is the main trauma center in Kashan and is governed by Kashan University of Medical Sciences. The inclusion criteria were having multiple trauma, being alive at hospital admission, and being transferred to the trauma center by EMS. All the patients who met the inclusion criteria were recruited and no one was excluded.

According to Barsuk et al. (20) and the protocols of EMS in Iran, all multiple trauma patients with head and chest injuries need to receive complementary oxygen during the pre-hospital phase and it was selected as the measure for necessity of oxygen therapy in the present study.

The study instrument consisted of three parts including a demographic questionnaire, the 6-item Trauma Assessment Questionnaire (TAQ), and the 4-item Oxygen Therapy Quality Assessment Questionnaire (OTQAQ) that were designed by the researchers. The demographic questionnaire consisted of four questions regarding patients’ age, gender, job, and education level. The 6-item TAQ included questions concerning the trauma occurrence date, type of trauma (blunt, penetrating, or both), the mechanism of trauma (traffic accident, fall, street fight, and debris fall), type of accident (pedestrian, bicycle, motorcycle, and car), and the time (day or night) and the place the trauma occurred. The OTQAQ assessed the quality of oxygen therapy during transferring patient to hospital and consisted of four items on performing lung auscultation, assessment of the rhythm of respiration, assessment of oxygen saturation, and administration of oxygen. The OTQAQ items were scored on a three-point scale in which 2 stood for ‘Done properly’, 1 for ‘Done improperly’, and 0 for either ‘Not done’ or ‘Not documented’. Accordingly, the total score of OTQAQ ranged from zero to eight. Then, the total score was divided by four (the number of questions) to make a criteria for measuring the quality of oxygen therapy. Consequently, scores lower and higher than two were interpreted as undesirable and desirable oxygen therapy, respectively. To validate oxygen therapy documentations, we also orally asked patients the following question, ‘Did you receive oxygen in ambulance?’ As far as unconscious patients were concerned, we were not able to ask them this question and hence, validated the administration of oxygen by searching for accompanying oxygen administration devices such as nasal cannula or facemask. We developed the study questionnaires based on an in-depth literature review. Then, we invited six nursing lecturers to assess the content validity of the questionnaires and their comments were included in the final version of the questionnaires. Content validity index (CVI) was calculated and was equal to 'one' as all experts agreed on the item relevance (23). moreover, the content validity ratio (CVR) was calculated using Lawshe method and it was equal to 'one' as all the experts agreed that all the items in the instrument were essential (24). To ensure the reliability of the instruments, we employed the inter-rater method. Accordingly, two raters administered the study questionnaires to ten patients. The inter-rater correlation coefficient was equal to 'one'. The Cronbach’s alpha was also calculated to be 0.79 using the data from 25 multiple trauma patients.

### 3.1. Data Analysis

Data analysis was performed using the Statistical Package for Social Sciences (SPSS v.16.0; SPSS Inc., Chicago, Illinois, USA). No missing value existed. All the data were described using frequency tables, central tendency measures, and variability indices. Moreover, Chi-square test was used to test the association between performing oxygen therapy and patients’ education level. Because Shapiro-Wilk test showed that the distribution of age was not normal, Mann-Whitney U test performed to examine the association between patients' age and receiving oxygen. In addition, logistic regression analysis was performed to predict the administration of oxygen in terms of factors such as place of accident, patients’ education level, time, and type of trauma.

### 3.2. Ethical Considerations

The study protocol was approved by the institutional review board with grant number 9206. Then, the study ethical considerations were approved by the Research Ethics Committee at Kashan University of Medical Sciences and issued by number 463 on May 4, 2013. We explained the aim of the study to the participants and ensured the confidentiality of their personal information. A verbal consent was obtained from the participants. In addition, data was collected after permission of the hospital and unit authorities. We also observed all ethical issues in accordance with the last version of the Helsinki Declaration.

## 4. Results

The study sample consisted of 263 (75.1%) male and 87 (24.9%) female patients. Participants' age ranged from two to 90 years, they were mostly (46.6%) between the age of 20 to 40 years with a mean of 34.19±18.71 years. Regarding education level, 86 (24.6%) patients were illiterate, 113 (32.3%) had elementary education, 71 (20.3%) had secondary education, and 58 (16.6%) were at high school level. In addition, 104 (29.7%) patients were workers among which 90 (86.53%) patients were industrial workers (Table 1). In total, 142 (40.6%) cases of all types of the trauma had happened during holidays. Moreover, 231 (66%) cases of all types of the trauma had happened in urban areas while 96 (27.4%) cases had happened in the country roads.

**Table 1.** Participants’ Occupational Status

Occupation	No. (%)
**Self-employment**	83 (23.70)
**Industrial worker**	90 (25.70)
**Homemaker**	65 (18.60)
**Student**	50 (14.30)
**Official worker**	14 (4.00)
**Other**	48 (13.71)

The most common mechanism of trauma was traffic accident that has occurred in 299 (85.4%) patients. Accordingly, motorcyclists, car passengers, and pedestrians constituted 52%, 20%, and 13.1% of the all trauma victims, respectively. Among the 252 car passengers and motorcyclists, 189 (75%) patients had not used safety equipment such as safety belt and helmet. Among all the trauma cases, 238 (68%) cases had happened during daytime. Moreover, 293 (83.71%) of the entire trauma victims had experienced both blunt and penetrating traumas, which were mainly (60%) on the head and neck area (Table 2). 

**Table 2.** Mechanism of the Trauma and Site of the Injury

Variable	No. (%)
**Mechanism of trauma**	
Accident	299 (85.40)
Fall	36 (10.30)
Street attack	11 (3.20)
Debris fall	4 (1.10)
**Site of injury**	
Head and neck	210 (60.00)
Upper extremities	228 (65.14)
Chest	41 (11.71)
Abdomen, back, and pelvis	113 (32.28)
Lower extremities	213 (60.85)

In total, 211 patients needed oxygen therapy during the pre-hospital phase; however, only 35 (16.58%) of them had received oxygen. None of the patients was assessed regarding oxygen saturation. Moreover, lung auscultation and rhythm of respiration were not documented in patients’ pre-hospital medical records. The OTQAQ total score for all patients was below 1, which denoted undesirable quality of oxygen therapy. By discarding the oxygen saturation assessment item that was performed for none of the patients we found that the quality of oxygen therapy has been desirable only for 16 (7.58%) of cases. On the other hand, in 176 patients (83.4%) whose pre-hospital medical records indicated the administration of oxygen, reported that they have not received oxygen therapy. Moreover, in 53.55% of all cases, oxygen therapy had not been documented at all (Table 3). 

**Table 3.** Documentation of Pre-Hospital Oxygen Therapy

Oxygen Therapy	No. (%)
**Not performed**	
Documented but not performed	66 (31.27)
Not documented and not performed	113 (53.55)
**Performed**	
Documented and performed	32 (15.16)
**Total**	211 (100)

Totally, 35 patients had received oxygen during the pre-hospital phase from which four patients were illiterate and the others were literate. Chi square test showed a significant association between oxygen therapy and mechanism of trauma (P = 0.018). In addition, Chi square test showed a significant association between oxygen therapy and patients’ education level (P = 0.023). The chance of administering oxygen was 3.39 times in literate patients (Odds ratio: 3.39, CI 95% 1.052-11.77). Moreover, out of 211 patients who needed oxygen, 72 patients were injured out of city (of them, 17 patients received oxygen) and 139 individuals were injured in the city (of them 18 received oxygen). Chi square test showed a significant association between oxygen therapy and place the trauma occurred (P = 0.048). The chance of administering oxygen was 2.078 times in patients who were injured out of city (Odds ratio: 2.078, CI 95%: 0.936-4.612). Moreover, 22 (62.85%) cases of oxygen therapy were performed during daytime and 24 (68.75%) cases were performed during working days of week. In addition, Chi square test showed significant association between mechanism of trauma and receiving oxygen (P = 0.018) so that from the total patients who received oxygen, 26 ones (74.3%) were injured due to traffic accident while others were injured due to fall, street attacks or debris. Moreover, Chi square test showed no significant relationship between gender (P = 0.09), job (P = 0.31), the day (P = 0.038), time of trauma (P = 0.31), type of accident (P = 0.56) and type of trauma (P = 0.49). Also, by using Mann-Whitney U test, no significant difference was observed between the mean age of the patients who received oxygen (32.51 ± 15.06) and those who did not receive it (35.51 ± 19.33) (P = 0.62, Z = -0.48).

Logistic regression analysis revealed that among all variables, the place of accident and patients’ education level were significant predictors for administration of oxygen during the pre-hospital phase (P < 0.001). These two variables were responsible for 3% to 6.4% of the variance of oxygen therapy (Table 4). 

**Table 4.** The results of Logistic Regression Analysis for Predicting the Administration of Oxygen in 211 Patients Who Had Indication for Receiving Oxygen

	B	S.E.	Wald	df	Sig.	Exp (B)	95.0% CI for EXP (B)	
							Lower	Upper
**Step 1**								
place of accident	1.270	0.607	4.376	1	0.036	3.560	1.083	11.696
education level	-0.483	0.216	4.983	1	0.026	0.617	0.404	0.943
Constant	1.291	0.650	3.943	1	0.047	3.636		

## 5. Discussion

The aim of this study was to investigate the quality of pre-hospital oxygen therapy in patients with multiple trauma. The study findings showed that most of patients with multiple trauma were males and between 20 to 40 years of age. This means that young males were at a higher risk for experiencing multiple trauma. This finding is in line with the findings of other studies (25-28). This is probably because of the fact that most of motorcyclists, cyclists, and truck drivers are men. Moreover, men usually choose hazardous technical jobs that put them at greater risk for experiencing trauma and injury.

Findings also revealed that the most common mechanism of trauma was road traffic accidents. We also found that accidents were more common among motorcyclists. Davoodabadi et al. also found that most of trauma patients were motorcyclists (29). In addition, Nguyen et al. (25) and Lin et al. (26) reported that the most common mechanism of trauma was accidents. However, Lin et al. reported that motorcycle accidents were the third leading cause of traumas (26) and Engel et al. found that only 9.3% of all the accidents were related to motorcycles (27). On the other hand, Engel et al. and Janssen and Burns found that the most common mechanism of trauma was fall (27, 28). Factors such as extensive use of motorcycles in Iran, particularly by reckless inexperienced young motorcyclists, and motorcyclists’ reluctance over wearing safety helmet contribute to the high prevalence of motorcycle accidents. Reluctance over using safety helmet and belt is an area of great concern. It not only can result in serious long-term disabilities, but also can increase health care costs. Consequently, the senior authorities, policymakers, and healthcare providers are responsible to develop effective strategies for decreasing the rate of accidents and preventing their complications.

Our findings also revealed that oxygen had been administered to only 16.6% of patients who needed it. However, the rate of oxygen therapy in studies conducted by Barsuk et al. (20) and Ahmadi-Amoli was about 80% (21). This statistics reveal the poor quality of pre-hospital oxygen therapy. Lack of an effective in-service education, supervision, and evaluation for the EMS staff might contribute to this finding. Haghparast-Bidgoli et al. have reported that paramedics’ lack of knowledge, experience, and mastery secondary to lack of continuing education programs-affects the quality of pre-hospital patient care negatively. Considering the importance of oxygen therapy in preventing long-term complications of trauma and injuries, development and implementation of effective in-service education programs in the area of oxygen therapy is recommended (5).

During the pre-hospital phase, patients’ need to oxygen therapy should be determined by monitoring blood oxygen saturation. However, although all of the ambulances are equipped with oxygen saturation monitoring devices, none of our participants was assessed in terms of oxygen saturation. This finding is in contrary with the results of Barsuk et al. (20) and implies that ambulance technicians have overlooked the importance of oxygen therapy. Again, effective in-service education programs are indicated here.

The findings of the present study indicated that 83.4% of the patients had not received oxygen during transferring to hospital setting. This finding implies that pre-hospital oxygen therapy provided to patients with multiple trauma was of low quality. A striking finding was that for 31.27% of these patients, the administration of oxygen had been documented in their pre-hospital medical records. This finding suggests that ambulance technicians have acknowledged the importance of pre-hospital oxygen therapy; however, they have been reluctant to perform it. Haghparast-Bidgoli et al. reported that as paramedics are paid poorly, they feel compelled to work in double or long shifts that in turn, sacrifice the quality of care (5). Given the importance of pre-hospital oxygen therapy and prevalence of road traffic accidents in our country, careful supervision and evaluation of pre-hospital care seems crucial.

Our findings revealed that literate patients were more likely to receive oxygen therapy during the pre-hospital phase. This finding might be attributed to the fact that, the number of literate patients was greater in this study. Moreover, we found that place of accident was another predictor of pre-hospital oxygen therapy. In other words, patients who experienced traumas at home, sport centers, or workplace were less likely to receive oxygen therapy in comparison to the victims of road traffic accidents. Probably, the longer distance between the place of accident and the hospital setting and the severity of road traffic accidents had affected paramedics’ decisions about administrating oxygen.

The quality of pre-hospital oxygen therapy provided to patients with multiple trauma was poor while these patients, particularly patients with chest traumas and head injuries, were in urgent need of oxygen therapy. Consequently, developing and implementing standard evidence-based protocols for oxygen therapy and administrating in-service education programs are recommended. To the best of our knowledge, this is the first study on the quality of pre-hospital oxygen therapy that was conducted in Iran. All data collections and observations in this study were conducted by the second author and this has decreased the possibility of inter-rater variations. However, the study was only conducted during four months and in one center. Therefore, the data might not necessarily mirror the performance of all EMS staff countrywide. Future and longer studies are needed t in different areas to portray a big picture of the performance of the pre-hospital emergency system in the critical aspects such as oxygen therapy in trauma patients. We investigated the quality of pre-hospital oxygen therapy in patients with multiple trauma. However, reasons for paramedics’ non-compliance with oxygen therapy standards remained unknown. Consequently, conducting studies on this subject area is recommended.
